# Exploration of the X-ray Dark-Field Signal in Mineral Building Materials

**DOI:** 10.3390/jimaging8100282

**Published:** 2022-10-14

**Authors:** Benjamin K. Blykers, Caori Organista, Matias Kagias, Federica Marone, Marco Stampanoni, Matthieu N. Boone, Veerle Cnudde, Jan Aelterman

**Affiliations:** 1 Pore-Scale Processes in Geomaterials Research Group (PProGRess), Department of Geology, Ghent University, 9000 Ghent, Belgium; 2 Ghent University Centre for X-ray Tomography (UGCT), 9000 Ghent, Belgium; 3 Swiss Light Source, Paul Scherrer Institute, 5232 Villigen, Switzerland; 4 Institute for Biomedical Engineering, University and ETH Zürich, 8092 Zürich, Switzerland; 5 Department of Physics and Astronomy, Ghent University, 9000 Ghent, Belgium; 6 Division of Engineering and Applied Science, California Institute of Technology, Pasadena, CA 91125, USA; 7 Environmental Hydrogeology, Department of Earth Sciences, Utrecht University, 3584 Utrecht, The Netherlands; 8 Image Processing and Interpretation, TELIN Department, Ghent University, 9000 Ghent, Belgium

**Keywords:** dark-field tomography, X-rays, mineral building materials

## Abstract

Mineral building materials suffer from weathering processes such as salt efflorescence, freeze–thaw cycling, and microbial colonization. All of these processes are linked to water (liquid and vapor) in the pore space. The degree of damage following these processes is heavily influenced by pore space properties such as porosity, pore size distribution, and pore connectivity. X-ray computed micro-tomography (µCT) has proven to be a valuable tool to non-destructively investigate the pore space of stone samples in 3D. However, a trade-off between the resolution and field-of-view often impedes reliable conclusions on the material’s properties. X-ray dark-field imaging (DFI) is based on the scattering of X-rays by sub-voxel-sized features, and as such, provides information on the sample complementary to that obtained using conventional µCT. In this manuscript, we apply X-ray dark-field tomography for the first time on four mineral building materials (quartzite, fired clay brick, fired clay roof tile, and carbonated mineral building material), and investigate which information the dark-field signal entails on the sub-resolution space of the sample. Dark-field tomography at multiple length scale sensitivities was performed at the TOMCAT beamline of the Swiss Light Source (Villigen, Switzerland) using a Talbot grating interferometer. The complementary information of the dark-field modality is most clear in the fired clay brick and roof tile; quartz grains that are almost indistinguishable in the conventional µCT scan are clearly visible in the dark-field owing to their low dark-field signal (homogenous sub-voxel structure), whereas the microporous bulk mass has a high dark-field signal. Large (resolved) pores on the other hand, which are clearly visible in the absorption dataset, are almost invisible in the dark-field modality because they are overprinted with dark-field signal originating from the bulk mass. The experiments also showed how the dark-field signal from a feature depends on the length scale sensitivity, which is set by moving the sample with respect to the grating interferometer.

## 1. Introduction

Our built patrimony consists largely of mineral building materials; that is, natural stones, clay-fired bricks, concrete, and mortars. These materials are used in almost every construction. As they are usually continuously exposed to changing weather conditions, causing material deterioration over time, masonry suffers from salt efflorescence after multiple wetting and drying cycles [1], giving the façade an aesthetically unpleasing white patina. Owing to nitrogen oxide compounds (NOx)—often originating from exhaust fumes—façades can grow a black crust [2]. On moist and shaded surfaces, microbial life can thrive, turning the stone green [3]. Repeated freezing and thawing can result in fractures in stones [4]. All of these processes have in common that they are (in)directly linked to water (liquid and vapor) migration in the pore space. This water dissolves minerals inside the building materials, transports salts to the stone surface, nurtures algae, and other microbial life, or—upon freezing—expands to ice resulting in high internal pressures exerted on the material.

The degree to which damage occurs is partially determined by pore space properties such as porosity, pore size distribution, and pore connectivity. Characterizing these properties is of primordial importance to understanding weathering processes. However, pore space characterization is a challenging field in itself as there is often a trade-off between sample size and the details the characterization method can obtain.

In the last 20 years, X-ray computed microtomography (µCT, micro-CT) has earned its place in the set of tools to investigate materials and their inner structures [5]. Micro-CT is based on the X-ray absorption by matter and can be performed both at synchrotron facilities and on lab-based systems. It generates a digital three-dimensional volume of the sample, consisting of a stack of two-dimensional grayscale images, in which the gray value represents the local linear absorption coefficient of the material in that voxel. Despite being a valuable tool to non-destructively investigate the inner structure of a material, like any other technique, it also has limitations: the quality of the material property analysis and the interpretation thereof is strongly dependent on the image properties such as resolution, signal-to-noise ratio, and the field of view. In practice, an important trade-off exists between the highest resolution at which a sample can be imaged and the field of view, which decreases at higher resolutions.

**Figure 1:**
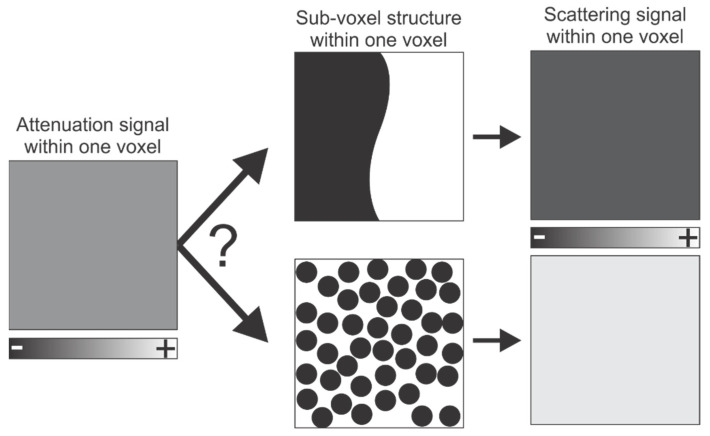
The same attenuation signal of a single voxel can be caused by different structures, as long as they have the same relative density (50% matter and 50% void in the given example). However, this different structure may result in a different scattering signal. This principle is what DFI is intended to exploit. In the attenuation signal, the − and + represent low and high attenuation, respectively. In the scattering signal, the − and + indicate less strong and stronger scattering, respectively.

X-ray dark-field imaging (DFI) is a modality intended to overcome some limitations of digital X-ray absorption imaging. It is a novel X-ray imaging modality based on measuring X-ray scattering by the sample rather than absorption [6]. This scattering occurs at structural heterogeneities in the sample that are smaller than the resolution. These heterogeneities are typically in the submicron regime. In a dark-field image, areas light up where this scattering occurs. DFI thus provides information on the sample that is complementary to the information provided by the (conventional) attenuation-based µCT image. If we consider a single voxels gray value, it is not possible to retrieve the underlying structure: two volumes with the dimensions of single voxel, consisting of 50% matter and 50% air, but with a different structure, would have the same attenuation signal. However, their distinct structure would result in a different scattering behavior (Figure 1).

X-ray scattering occurs at internal heterogeneities, tens of nanometers in size up to several micrometers [6,7]. Taking into account that, in attenuation-based CT, a feature should be three to ten times larger than the spatial resolution of the image to be positively identified (Figure 1), these micro- and nanoscopic heterogeneities typically remain below the resolution of a µCT system (around 1 µm for laboratory-based systems and several hundreds of nanometers for synchrotron-based systems) [5,8]. In a dark-field image, zones with scattering heterogeneities cause strong signal values, which is hypothesized to be indicative of features in the sample unresolved by attenuation-based imaging (e.g., cracks, inclusions, and pores). DFI thus provides information about features below the attenuation imaging resolution limit of a particular detector, thereby (at least partially) mitigating the trade-off between resolution and field-of-view [5]. This also implies that, when two materials have similar bulk properties and are not discernable using standard X-ray attenuation—which is mainly sensitive to composition and density [5]—they might be distinguished using X-ray dark-field imaging, provided that they have a different unresolved microstructure [9].

In 2008, dark-field imaging was performed for the first time with a grating interferometer (GI) [6], which is also the approach used in this study. Conceptually, this interferometer generates an interference pattern that is changed by the presence of a sample. From this change, the dark-field signal can be retrieved (along with differential phase contrast and attenuation contrast).

**Figure 2:**
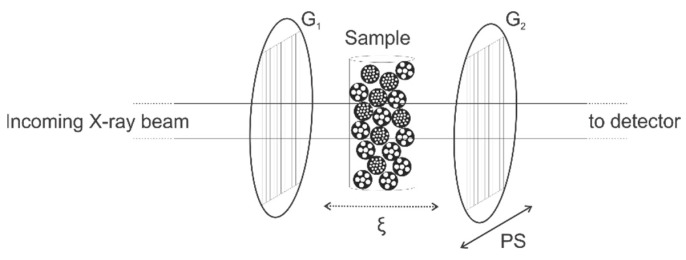
Schematic setup of a grating interferometer in a parallel X-ray beam. To change the correlation length (ξ), the sample is moved in the beam direction. Phase stepping (PS) is done by movement of G2, parallel to G1, perpendicular to the beam direction [9].

This interferometer consists of three gratings: a source grating (G_0_), a phase grating (G_1_), and an analyzer absorption grating (G_2_). G_0_ is an optional grating used to create spatial coherence of the X-ray beam when large (i.e., millimeter-sized) X-ray focal spots are used. The phase grating (G_1_) generates an interference pattern at discrete distances from G_1_ (i.e., Talbot distances), which is sampled by G_2_. G_2_ has absorbing lines and the same orientation as G_1_, and is placed right in front of the detector (Figure 2). Different setup geometries exist: the sample can either be placed in front (upstream) of G_1_ and G_2_ or between G_1_ and G_2_; however, the principle is the same [10].

**Figure 3:**
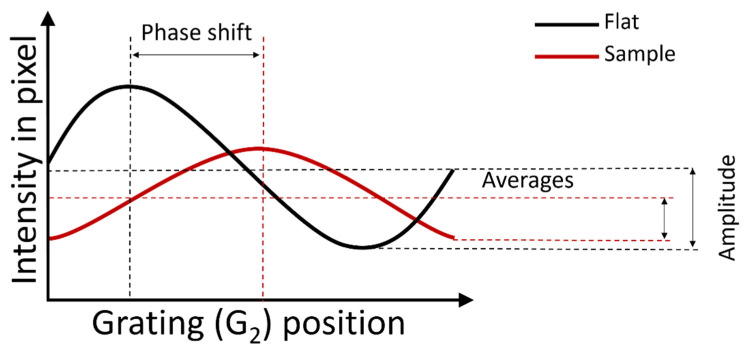
Phase stepping G_2_ results in an intensity oscillation, which is used to extract attenuation (average intensity), phase-contrast (phase shift), and dark-field images (intensity amplitude).

As G_2_ moves parallel to G_1_, and perpendicular to the orientation of the grating lines and the beam direction, an intensity oscillation in the detector pixels is generated. This movement of G_2_ is called phase stepping (PS). The resulting oscillation, which is expressed as intensity amplitude over distance moved by G_2_, is called the phase stepping curve (Figure 3) [6,7,11,12,13]. When phase stepping is performed both with and without a sample in place, the average intensity, amplitude, and phase of this oscillating function will be different. These changes may be quantified after extracting absorption images (average intensity), differential phase contrast images (phase shift), and dark-field images (intensity amplitude), respectively.

The ratio of the intensity amplitude to the average intensity is called the fringe visibility V and the decrease in intensity amplitude when a sample is inserted is called the visibility reduction $V^{r}.$ The dark-field signal is then described quantitatively as follows [10,14,15]:

(1) $$-\log\left(V^{r}\right)=-\log\left(\frac{V_{sample}}{V_{flat}}\right)=-\log\left(\frac{Average_{flat}\;Amplitude_{sample}}{Average_{sample}\;Amplitude_{flat}\;}\right)\;$$

This visibility reduction is caused by heterogeneities inside the sample’s voxels affecting the interference pattern formation; this reduction makes up the dark-field signal [16]. In practice, however, the visibility reduction is not solely caused by intra-voxel heterogeneities: crosstalk between different detector pixels and partial volume effects also contribute to the dark-field signal. It should also be noted that image artifacts that commonly occur in absorption images, e.g., beam hardening, also affect the dark-field modality [13,17,18,19,20]. These artifacts make it challenging to extract meaningful information from the dark-field signal.

Yashiro et al. (2010, 2011) and Lynch et al. (2011) have laid the foundations of quantitatively linking the dark-field signal to sample characteristics such as average feature size (in the plane of the grating, in the direction perpendicular to the grating lines) by defining the correlation length (*ξ*) [10,21,22].

The correlation length (*ξ*) can be regarded as the length-scale sensitivity of the setup [10]. At a synchrotron beam line (parallel beam geometry and monochromatic X-rays), the correlation length is defined by following formula:

(2) $$\xi =\frac{\lambda_{eff}L_{s}}{p}\;$$

with $\lambda_{eff}$ is the effective energy of the system, $L_{s}$ is the distance between the sample and the detector, and p is the grating pitch [11].

In a parallel beam geometry, the correlation length is changed by moving the sample closer to or further from the detector (Figure 2).

### Applications of X-ray Dark-Field Imaging (DFI)

As grating-based X-ray dark-field imaging is still not a widely available method, the acquisition of satisfactory dark-field images is far from straight-forward. However, with the dark-field signal being more characterized and formalized in the literature, more applications of X-ray dark-field are being published as well. Two of the first and most explored applications of X-ray DFI are mammography and lung imaging [23,24,25,26,27,28].

DFI has found applications in the food industry (DFI is sensitive to the raw, frozen, and defrosted state of fruits [29] and can monitor the germinating of barley seeds, providing valuable insights into the growth process [30]), material science (DFI has enabled the study of pores and defects in fiber-reinforced polymers [31,32,33,34,35], composites [36,37], stearin [38], thermoplastics [39], and metals [40,41], invisible in standard attenuation-based imaging because of their size or composition), and the building industry (monitoring of hardening of cement [42,43] and detection of micro-cracks in concrete [44]).

All of these applications have in common that they consist of either a scattering object in soft tissue or structurally rather homogeneous materials. To the best of our knowledge, there is no published research on X-ray dark-field tomography of structurally complex materials like mineral building materials with the aim to characterize sub-resolution features.

In this manuscript, we present an investigation into the dark-field signal generated by mineral building materials. Samples from natural sandstone, a fired clay brick, a roof tile, and a carbonated mineral building stone were scanned at the TOMCAT beamline of the Swiss Light Source (Paul Scherrer Institut, Villigen, Switzerland) using grating interferometry (GI) for DFI. To aid the interpretation of the dark-field images, high-resolution images were also acquired at this facility.

## 2. Methods and Materials

### 2.1. Grating Interferometry-Based X-ray Dark-Field Tomography and High-Resolution µCT

Grating interferometry-based X-ray dark-field µCT was performed at the TOMCAT beamline of the Swiss Light Source synchrotron of the Paul Scherrer Institut (Villigen, Switzerland) [45]. The grating interferometer (GI) was a π phase shift interferometer, consisting of a linear silicon phase grating with a pitch of 4 µm and depth of 32 µm, as well as a linear gold analyzer grating with a pitch of 2 µm with a depth of 50 µm. The isotropic voxel size was 6.5 µm (magnification 1×).

A 300 µm LuAG/Ce scintillator was used together with a pco.edge 4.2 camera [46]. For all of the scans performed with the GI, the beam energy was 25.0 keV and the exposure time per projection 160.0 ms.

The GI was operated in the third fractional Talbot order, with a distance of 12 cm between the two gratings. The sample was rotated over 180°. Per correlation length (CL), 10 dark images were acquired and the phase stepping curve was constructed based on eleven phase steps, each starting with 50 open beam images. The dark images and open beam images serve to correct the projections for background noise and detector errors such as dead pixels. The image acquisition settings per sample are shown in Table 1.

High-resolution tomograms were acquired using a 20 μm LuAG/Ce scintillator together with a pco.edge 5.5 camera and a 10× objective. A total of 3001 projections were taken over 360°, as well as 10 dark images and 50 flat-field images. The scanning energy was 21.00 keV and the exposure time 500 ms. The isotropic voxel size in the reconstructed tomograms was 0.65 μm.

The absorption- and dark-field projections were reconstructed to 3D volumes with the gridrec algorithm [47]. The high-resolution tomograms were reconstructed with an additional ring removal algorithm with a wavelet/FFT-based routine [48].

### 2.2. Bray Sandstone

**Figure 4:**
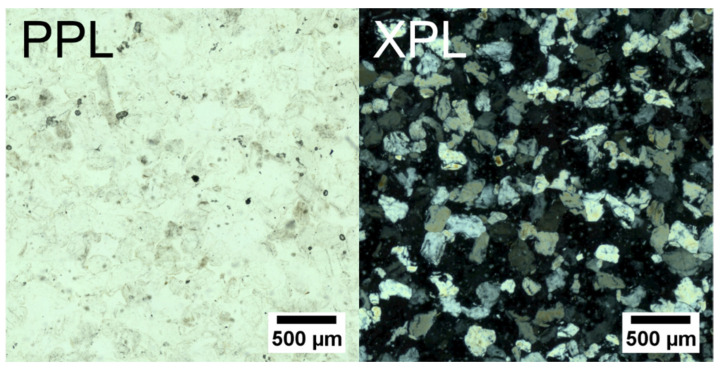
Thin section photographs of the Bray sandstone under plane- and cross-polarized light (PPL and XPL, respectively). The stone consists entirely of quartz grains. The difference in their lattice orientation results in different shades of gray under cross-polarized light. Adapted from Flepostore—https://flepostore.ugent.be/rock/be-22-0017, accessed on 9 October 2022.

The Bray sandstone is a Belgian quartz arenite from the Paleocene. It consists of irregular concretions of quartz grains with a siliceous cement. It has been used as a construction stone in several buildings in the Province of Hainaut, Belgium [49]. A thin section photograph in both plane- and cross-polarized light is shown in Figure 4.

**Figure 5:**
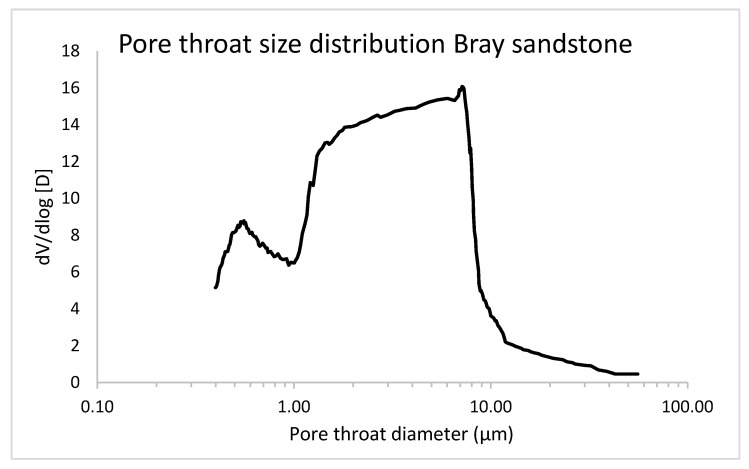
Pore size distribution of a sample of Bray sandstone, determined using MIP.

The pore size distribution was determined with mercury intrusion porosimetry (MIP) on a sample of 8 mm in diameter using a Pascal 440 mercury intrusion porosimeter (Thermo Fisher Scientific, Waltham, MA, USA). In Figure 5, the pore throat size distribution is shown, indicating that 97.30% of the pore volume has pore throats larger than 1000 nm. However, a smaller peak can also be observed between pore throat sizes of 400 and 1000 nm. This smaller range is mainly interesting for investigation with DFI, as these are below the resolution of most conventional µCT systems. Based on the MIP measurement, the total open porosity was only 4.9%. The Bray sandstone was considered an interesting case study for DFI because of its homogeneous composition and structure.

### 2.3. Ceramic Samples

A standard fired clay brick (TB) and a roof tile (TL) were selected as interesting study samples for DFI, as they are considered to be very challenging to characterize using absorption-based µCT because of their pore size parameters. They are known to have pore sizes smaller than the voxel size in standard X-ray CT absorption images (in the order of several micrometers). Both the fired clay brock (TB) and the roof tile (TL) were characterized using mercury intrusion porosimetry (MIP), using the AutoPore IV 9500 porosimeter (Micromeritics instrument corporation). MIP was performed on a cylindrical TB sample with a diameter of 14 mm and length of 40 mm. The MIP sample had a diameter of 14 mm and a length of 12 mm. Both samples were measured twice. Based on the MIP results, the TB and TL samples had an open porosity of 35.69 ± 0.17% and 35.12 ± 0.11%, respectively.

**Figure 6:**
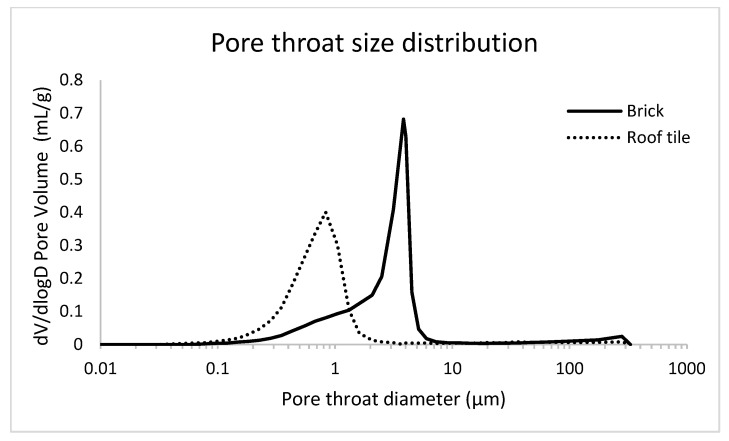
Pore throat size distribution for the ceramic samples, based on MIP.

In Figure 6, the pore throat size distribution of both the fired clay brick and the roof tile samples is given. Sample TB has a mode pore throat diameter of around 4 µm, with pore throats ranging down to as small as 0.1 µm. For roof tile (TL), the mode of the pore throat diameter lies around 0.8 µm and almost no pore throats are measured with a diameter exceeding 2 µm.

It must be noted that these are measurements of the pore throat diameter, the smallest part of the pore. Although it is only a partial measurement of the pore dimensions, it is still indicative of the presence of features below the image resolution (voxel size of 6.5 µm).

Additionally, the pore space was visualized using scanning electron microscopy (SEM) and conventional absorption µCT. All SEM images were acquired using a MIRA3 TESCAN scanning electron microscope equipped with a field emission gun (FEG) at a scanning voltage of 15.0 kV and an isotropic pixel size of 979.40 nm. Conventional absorption µCT was performed at the TOMCAT beamline of the Swiss Light Source.

**Figure 7:**
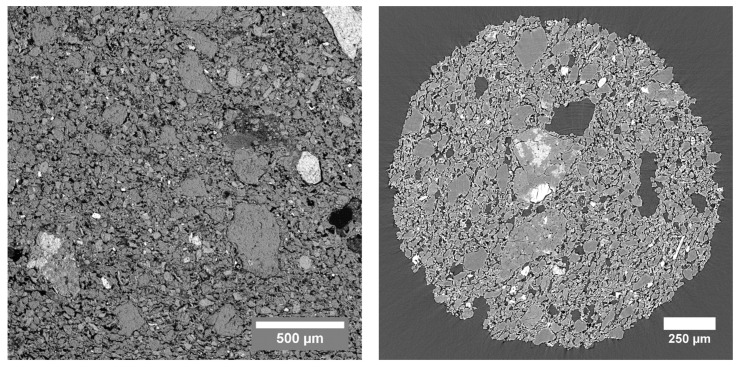
(**Left**): SEM image of the TB sample, acquired with back-scattered electrons at a working distance of 15.28 mm. (**Right**): Cross section through the absorption-µCT dataset of the TB sample acquired at the TOMCAT beamline.

In Figure 7, a back-scattered electron image of a thin section of the TB sample is shown together with a µCT image. Gray value segmentation based on simple thresholding of the pores in the SEM image resulted in a total porosity of 25.34%. In the three-dimensional µCT dataset, the same approach of gray value segmentation resulted in a measured total porosity of 32.00%. The difference in these two values could be attributed to the fact that these measurements were not on the same sample, and on a volume that is too small to be representative for the whole material [50].

After checking whether the signal-to-noise ratio was still sufficient, the number of projections per phase step was decreased to 501 to lower scanning durations.

**Figure 8:**
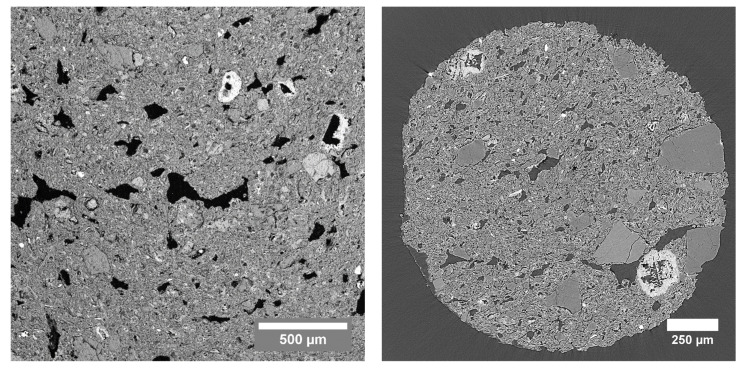
(**Left**): SEM image of the TL sample, acquired with back-scattered electrons at a working distance of 15.08 mm. (**Right**): Cross section through the µCT dataset of the TL sample acquired at the TOMCAT beamline.

In Figure 8, a thin section SEM image of the TL sample is shown together with a µCT image. Gray value segmentation of the SEM image resulted in a total porosity of 23.91%. In the three-dimensional µCT dataset, a total porosity of 34.00% was obtained.

### 2.4. Carbonated Mineral Building Material

Mineral carbonation is a naturally occurring chemical weathering process during which oxide bearing minerals are transformed to more stable, insoluble carbonates. In an industrial context, this process is accelerated from millions of years to a matter of hours, and can be used to create CO_2_-negative building materials. A general reaction of these metal (Me)-bearing silicates with CO_2_ is as follows [51,52]:

(3) $${\mathrm{Me}}_{x}{\mathrm{Si}}_{y}O_{x+2y-t}{\left(\mathrm{OH}\right)}_{2t}+x\;{\mathrm{CO}}_{2}⇌x\;{\mathrm{MeCO}}_{3}+y\;{\mathrm{SiO}}_{2}+t\;H_{2}O\;$$

To create building stones, steel slags are ground into small grains, rich in Ca and Mg (either in the form of silicates or as oxides). These grains are wetted and compacted into a brick shape (regular, stackable). After compaction, the hydration will start. During this hydration, earth alkaline phases will dissolve into the water, freeing OH^−^-ions into the water, increasing the pH. After hydration, the building blocks are placed in an autoclave, exposed to a high pressure CO_2_ environment and at high temperatures. The CO_2_ will dissolve in the water between the grains and form HCO_3_^−^ and CO_3_^2−^, lowering the pH, allowing the dissolution of Ca-silicates, leaving a Ca-depleted amorphous silica rim in the grain. The dissolved Ca^2+^ will react with the CO_3_^2−^ to form CaCO_3_. The hydrated portlandite (Ca(OH)_2_) will also react with CO_2_ to form calcite and water. The precipitation of calcite binds the grains together in the block and generates strength [52].

The carbonated building material used in this research consisted of a mixture of 65% quartz grains (grain size 0–1 mm) and 35% mineral steel slag residue (grain size 0–0.5 mm). Based on water absorption porosimetry under vacuum, an open porosity of 26.34% was obtained.

**Figure 9:**
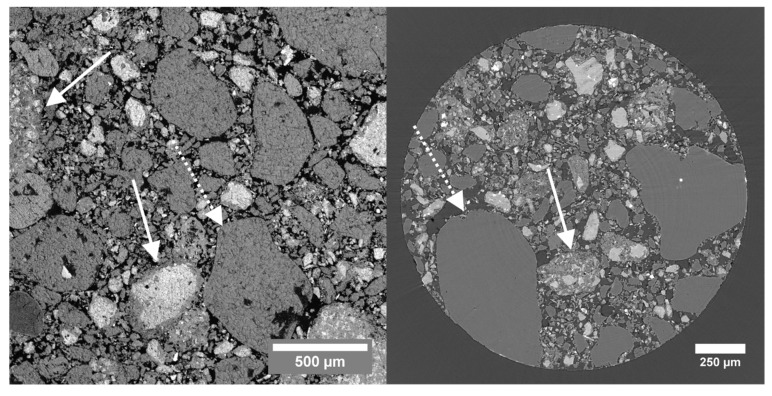
(**Left**): Thin section SEM image of the carbonated sample, acquired using back-scattered electrons, at a working distance of 15.18 mm. (**Right**): Cross section through the µCT volume, acquired at TOMCAT. The solid and dashed arrows indicate carbonated matter and quartz grains, respectively.

Figure 9 on the left shows a thin section SEM image of the carbonated sample, acquired with back-scattered electrons. On the right, a cross section through a µCT volume, acquired at the TOMCAT beamline, is shown. The dashed arrow indicates a quartz grain; the solid arrows indicate calcite grains. The quartz grains show a more homogeneous gray value than the carbonate fragments. Segmenting the pores based on their gray value in the SEM image resulted in a total porosity of 27.50%, while the gray value segmentation of the pores in the µCT volume resulted in a porosity of 26.45%. Both of these porosities are very similar to the 26.34% obtained using water absorption. The sample was too brittle for MIP measurements. As this sample consisted of both very homogeneous and very heterogeneous grains, the sample was considered to be an interesting study case to explore with DFI. After embedding the sample in resin (Struers EpoFix Resin with EpoFix Hardener), a sample with a diameter of 1.5 mm was drilled.

## 3. Results

### 3.1. Bray Sandstone

**Figure 10:**
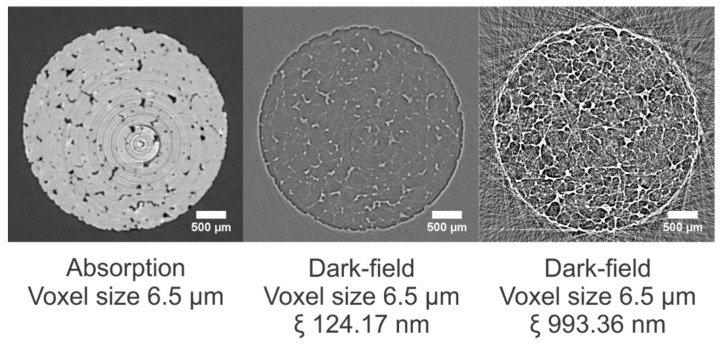
Cross section through the absorption dataset of a 3 mm Bray sample and corresponding cross section through the dark-field datasets at correlation lengths of 124.17 and 993.36 nm. The dark-field images have equal gray value scaling.

The Bray sandstone is a pure quartzite and, structurally, this building material is quite homogeneous (see Section 3.2). This is confirmed in the dark-field modality (Figure 10); that is, the sample exhibits low dark-field signal values inside the material (homogenous material). Most scattering is observed at the outer edge of the sample and along the grain boundaries, where the difference in refractive index (between air and the quartz grains, consisting of SiO_2_) is the largest. At a correlation length of 993.36 nm, scattering along the edges causes saturation of the signal.

### 3.2. Brick (TB)

**Figure 11:**
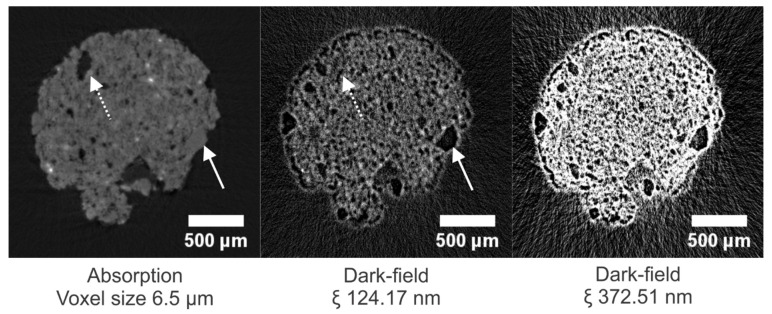
Cross sections through the absorption dataset and corresponding dark-field datasets of the TB sample at different correlation lengths. The image voxel size is 6.5 µm. The solid and dashed arrows indicate a quartz grain and a large pore, respectively. The dark-field images have equal gray value scaling.

In Figure 11, cross sections through the absorption dataset and corresponding dark-field datasets at CLs of 124.17 nm and 372.51 nm, respectively, are given (additional correlation lengths are shown in Supplementary Figure S1). In the absorption image, a quartz grain (indicated with a solid white arrow) is almost indistinguishable from the porous matrix based on its X-ray attenuation. However, having a homogeneous structure, a quartz grain causes less X-ray scattering in comparison with the porous fired clay bulk mass of the sample. Therefore, in the dark-field image, this bulk mass is observed to appear much brighter than the quartz grain. Large pores, on the other hand, which appear black in the absorption image (indicated with the dashed arrow), were also expected to cause no scattering and show no signal in the dark-field image. However, this is not the case. A possible explanation is a strong scattering caused by the pore-to-air interface determining the dark-field signal. At this location, scattering is caused by both the large density difference between matter and air and by surface roughness.

### 3.3. Roof Tile (TL)

**Figure 12:**
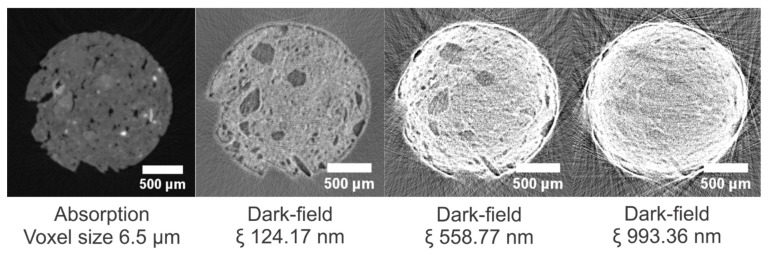
Cross sections through the absorption dataset and corresponding dark-field datasets of the TL sample at three correlation lengths, ranging from 124.17 to 993.36 nm. The dark-field images have equal gray value scaling.

In Figure 12, cross sections through the absorption dataset and corresponding dark-field datasets are given. The quartz grains in the material are difficult to distinguish in the absorption image, but are clear in the dark-field image owing to their homogeneous structure, whereas the pores can be seen in the absorption mode, but are masked in the dark-field mode. In Supplementary Figure S2, cross sections at additional correlation lengths are shown.

From a correlation length of 310.43 nm onwards to greater distances, the edges of the sample become increasingly bright, indicating the saturation of the dark-field signal, i.e., a vanishing of the dark-field visibility with the sample present (Figure 3 and Figure S2).

**Figure 13:**
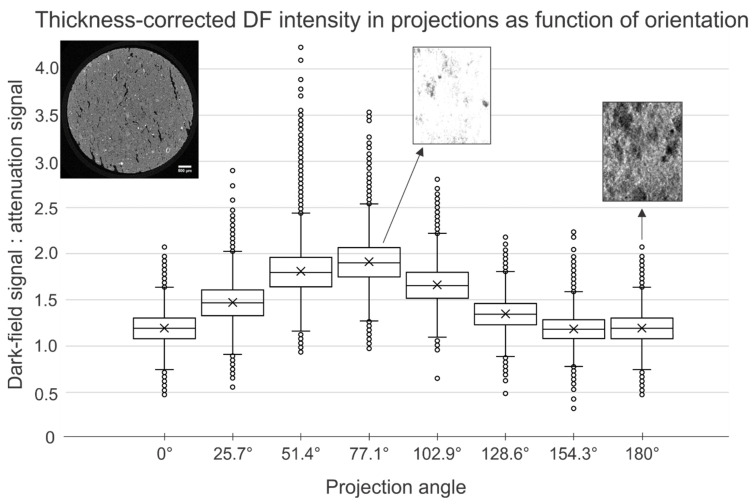
Measured intensity in the extracted dark-field projections, cropped to contain the center part of the sample. At certain angles, the dark-field signal is much stronger, indicating an anisotropy in the sample structure. This can be attributed to the compression of the clay in the production process, resulting in anistropic pores (flat instead of spherical). Two dark-field projections (with equal scaling) corresponding to angles of 77.1° and 180° are included, showing the visible saturation of the dark-field signal. In the upper left corner, a cross section through an absorption tomogram is shown, indicating the anisotropic pore structure (acquired at UGCT with a voxel size of 4 µm).

When the dark-field projections were investigated, it was noted that the scattering intensity (observed as brightness) was not uniform over the projections (acquired over 180°). In Figure 13, the image intensity in the sample was plotted for eight projections evenly spread over the 180°. The observation of angle-dependent dark-field signal, indicative of angle-dependent scattering, could be explained by the anisotropic structure inside the TL sample. In contrast to the fired clay brick TB, the TL sample was compressed using a press. This results in flattened pores instead of isotropic pores. This anisotropy can already be seen in µCT (Figure 13, upper left).

Linear gratings are sensitive to features perpendicular to the grating lines [53]. At angles where the anisotropic pores are parallel to the grating lines, the DF signal is higher than at other angles.

### 3.4. Carbonated Sample

The carbonated sample consisted of 65% of quartz grains with grain sizes between 0 and 1 mm, and of 35% of the mineral fraction of steel slags with grains sizes between 0 and 0.5 mm.

**Figure 14:**
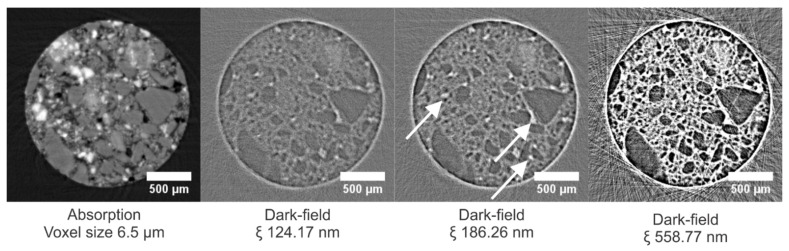
Cross sections through the absorption dataset and corresponding dark-field datasets of the carbonated sample. The dark-field images have equal gray value scaling. Bright spots of scattering at the lower CLs correspond to pores not (completely) filled with the resin. Large quartz grains scatter less, whereas the steel slag residue has an in-between scattering behavior. The white arrows indicate local scattering zones, corresponding to pores that were not completely filled with resin.

**Figure 15:**
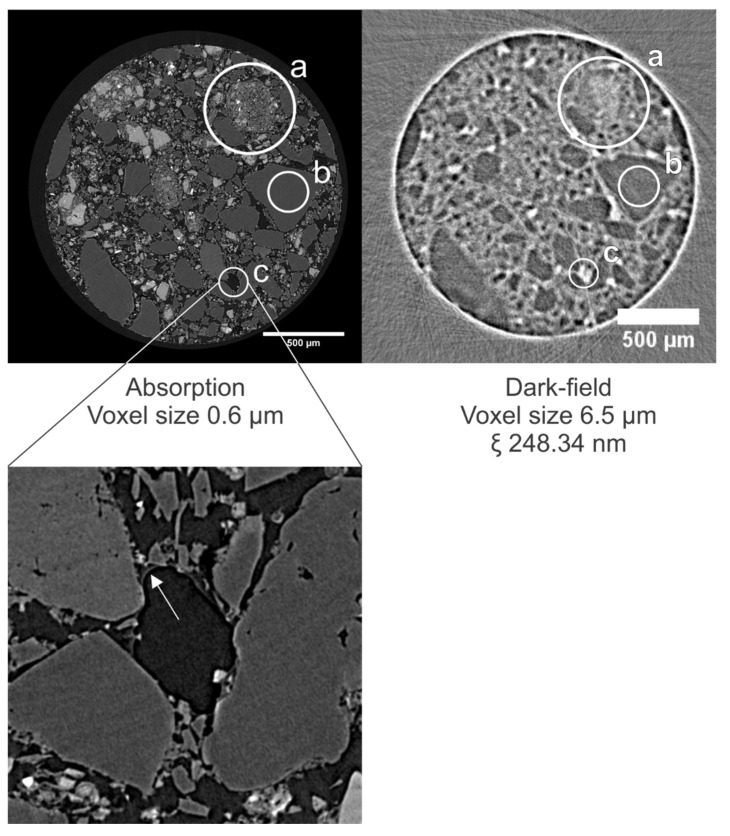
(**Top left**): Cross section through the high-resolution tomogram of the carbonated sample. (**Top right**): Cross section through the dark-field tomogram at the same height in the sample as in Figure 14. The steel slag residue (a) scatters X-rays more than the quartz grains (b). Localized regions of scattering correspond to pores that are not completely filled with resin (c). (**Bottom**): Zoom on a pore where the resin has not filled the entire pore. The edge of the resin is indicated with an arrow. The arrow itself is located in the air-filled pore.

In Figure 14, a cross section through the absorption dataset of the sample and corresponding dark-field datasets at different correlation lengths is shown (cross sections through tomograms at additional CLs are shown in Figure S3). In the absorption tomograms, the quartz grains are uniformly gray, whereas the brighter gray values are caused by the steel slag residue. In the dark-field modality, the quartz grains can be distinguished easily by a low DF signal, as was the case in the brick (Figure 15, zone b). Grains of steel slag residue, having a heterogeneous composition, scatter X-rays, but the edges of the grains can hardly be distinguished in the dark-field modality (Figure 15, zone a). The cross sections in the dark-field tomograms at different correlation lengths in Figure 15 also show isolated regions that scatter more X-rays than their surroundings (Figure 15, zone c). Zooming in on these regions in the high-resolution tomograms (voxel sizes of 0.65 µm) indicated that these regions correspond to pore spaces that were not filled with resin, leaving a high difference in the refractive index between the air and the (hardened) resin. Combined with the surface roughness at the edge of the grains, this difference in the refractive index results in high amounts of X-rays being scattered at these locations. These local scattering zones are more distinguishable at lower correlation lengths (indicated in Figure 14 with white arrows at a CL of 186.26 nm); at higher correlation lengths (in Figure 14 at a CL of 558.77 nm), these zones are masked by scattering from other sources (e.g., grain boundaries, smaller pores).

## 4. Discussion and Conclusions

Dark-field tomography generates a contrast based on sub-voxel-sized structural features in a sample, a material property inaccessible to attenuation-based tomograms, thus DF tomograms complement conventional attenuation-based tomograms. This is most clear in the ceramic samples: the quartz grains are hard to distinguish in the attenuation tomograms, but clear in the dark-field tomograms. However, it was not possible to extract information on the unresolved pore space of the samples. This can be attributed to the frequent saturation of the DF signal and overprints of the pore signals by the strong scattering occurring at the edges of pores and other unresolved features.

In both the fired clay samples and the carbonated sample, the edges of macroscopic (i.e., resolved) features in the material scatter heavily, obscuring potentially interesting scattering originating from features below the resolution scale. This bulk scattering could be attributed to surface roughness, small pores below the resolution of the image, and/or the large difference in the refractive index between air and matter. Low scattering was observed within the quartz (SiO_2_) in the fired clay materials, mineral building materials, and Bray sandstone alike.

In all cases, the contrast-to-noise ratio between strongly and weakly scattering parts was best at the lowermost correlation lengths, as at the higher CLs, the visibility is often reduced to such a degree that sensitivity is lost, in a process called dark-field saturation. This saturation also determines the feasible sample size. For example, we were able to scan the Bray sandstone, only exhibiting scattering at the edges, at a sample size of 3 mm without dark-field saturation. For the fired clay bricks and carbonated building materials, there are too many scattering features in a 3 mm sized sample, resulting in saturation. Even at a sample size of 1.5 mm, the dark-field signal became saturated at correlation lengths of 248.34 nm and higher.

The third fractional Talbot order (TO3) was chosen because it allowed to scan a larger range of correlation lengths. However, dark-field sensitivity may be improved by scanning in Talbot order (TO) 1 instead of TO 3; when the distance between the gratings is reduced, the SNR increases with the visibility for a Talbot Interferometer [54]. In TO 1, the intergrating distance $d_{G_{1}G_{2}}$ would be 4 cm instead of 12 cm. Alternatively, the sample can be embedded in a different material, one that is chosen to lower the difference in refractive index between the sample and its surroundings. The easiest medium would be water. Water lowering the scattering of X-rays has been described in the works of Yang et al. (2014, 2018). In the carbonated sample, the pore space was filled with epoxy resin. In the dark-field tomograms (Figure 15), we can barely distinguish the pore space from the bulk material. It is, however, surprising that the resin, which is not crystalline, scatters X-rays. This scattering might be due to unresolved air bubbles or impurities trapped in the resin.

### 4.1. Anisotropic Sample Structures

In the roof tile (TL) sample, the saturation with scattering was dependent on the projection angle. We assume that these projections correspond to sample orientations in which the plane of flattened pores was parallel to the X-ray beam, perpendicular to the grating plane (Figure 13). The inconsistent signal in the projections also resulted in more artefacts in the reconstructed tomograms (Figure 12 and Figure S2). For a sample drilled perpendicular to the flattened pores, this anisotropy would not be in a plane containing the rotation axis, and the scattering intensity of the sample would not vary depending on the angle of rotation. However, it was not possible to drill and scan such a sample during the allocated beam time. It is worth noting that other methods exist that are well-suited for anisotropic sub-voxel structures, such as X-ray scattering tensor tomography [35] and the use of gratings with circular unit cells [32].

### 4.2. Scanning Durations

DFI scanning durations are typically much longer than when performing conventional µCT. This is because of the need to perform a full scan per phase step, multiplying the conventional scanning time by the number of phase steps (typically a minimum of five to plot the phase stepping curve (Figure 3)). If one wishes to perform DFI at multiple correlation lengths, scanning durations must be multiplied by the number of desired correlation lengths. It must also be noted that advances have been made towards single-shot DFI that no longer requires phase-stepping [55,56,57].

### 4.3. Outlook

With the limited published research on dark-field tomography on geomaterials, choosing the correlation length (and Talbot order) at which to scan is currently still a process of trial and error. Moreover, all geomaterial samples used in this research had a diameter of 1.5 to 3 mm, which is smaller than part of the grains or pores in the respective materials and renders the samples not representative. As larger samples would currently still saturate the DF signal, at least a large number of samples should be scanned to study the influence of the material’s heterogeneity on the DF signal and objectively link a DF signal to the material. This should be done for the entire range of correlation lengths possible.

For the investigated materials (natural sandstone, fired clay bricks and roof tiles, and carbonated materials), the best results (in the third fractional Talbot order) can be achieved at correlation lengths ranging up to 372.51 nm. At higher correlation lengths, dark-field signal saturation occurs, which is detrimental to the quality of the reconstructed tomograms. In the first fractional Talbot order, this saturation might be acting up at higher correlation lengths. However, there is currently no proof that tomograms acquired at these higher correlation lengths would yield additional information on the samples’ microstructure.

Ideally, a database should be created, containing as many material properties as possible, as well as scanning parameters and the resulting signals, allowing users to choose optimal scanning parameters. It is advisable, however, to start this database with simple materials, such as the non-porous elementary units of (building) stones: pure quartz (SiO_2_), calcite (CaCO_3_), alumina (Al_2_O_3_), and so on. Subsequently, different, known porosities and pore sizes should be included in these ‘reference measurements’. This would ultimately allow to extract quantitative information such as the sample’s unresolved porosity and/or pore size using dark-field imaging. Vice versa, this knowledge would allow to scan at interesting correlation lengths based on prior knowledge coming from, e.g., MIP.

## Figures and Tables

**Table 1:** Overview of at which correlation lengths the different samples were scanned. TB and TL are a fired clay brick and roof tile, respectively; the carbonated material is abbreviated as carb. Bray is a natural sandstone. X indicates whether tomograms were acquired at that correlation length.

Correlation Length (nm)	Sample-G_2_ Distance (mm)	Sample			
		TB	TL	Carb.	Bray
124.17	10	X	X	X	X
186.26	15	X	X	X	
248.34	20	X		X	
310.43	25		X	X	
372.51	30	X		X	
434.60	35		X	X	
496.68	40			X	
558.77	45		X	X	
682.94	55		X		
807.11	65		X		
931.28	75		X		
993.36	80		X		X
Projections per phase step		501	1001	501	1001
Sample diameter (mm)		1.5	1.5	1.5	3

## Data Availability

The data presented in this study are available on request from the corresponding author.

## Supplementary Materials

The following supporting information can be downloaded at https://www.mdpi.com/article/10.3390/jimaging8100282/s1, Figure S1: Cross sections through the absorption dataset and corresponding dark-field datasets of the TB sample at different correlation lengths. The image voxel size is 6.5 µm. The solid and dashed arrows indicate a quartz grain and a large pore, respectively. The dark-field images have equal gray value scaling. Figure S2: Cross sections through the absorption dataset and corresponding dark-field datasets of the TL sample at nine correlation lengths, ranging from 124.17 to 993.36 nm. The dark-field images have equal gray value scaling. Figure S3: Cross sections through the absorption dataset and corresponding dark-field datasets of the carbonated sample. The dark-field images have equal gray value scaling. Bright spots of scattering at the lower CLs correspond to pores not (completely) filled with the resin. Large quartz grains scatter less, whereas the steel slag residue has an in-between scattering behavior. The white arrows indicate local scattering zones, corresponding to pores that were not completely filled with resin.
