# Supplementary figures and images for: Exploration of the X-ray Dark-Field Signal in Mineral Building Materials
Source: J Imaging. 2022 Oct 14;8(10):282. doi: 10.3390/jimaging8100282 (PMC9604867; doi:10.3390/jimaging8100282)

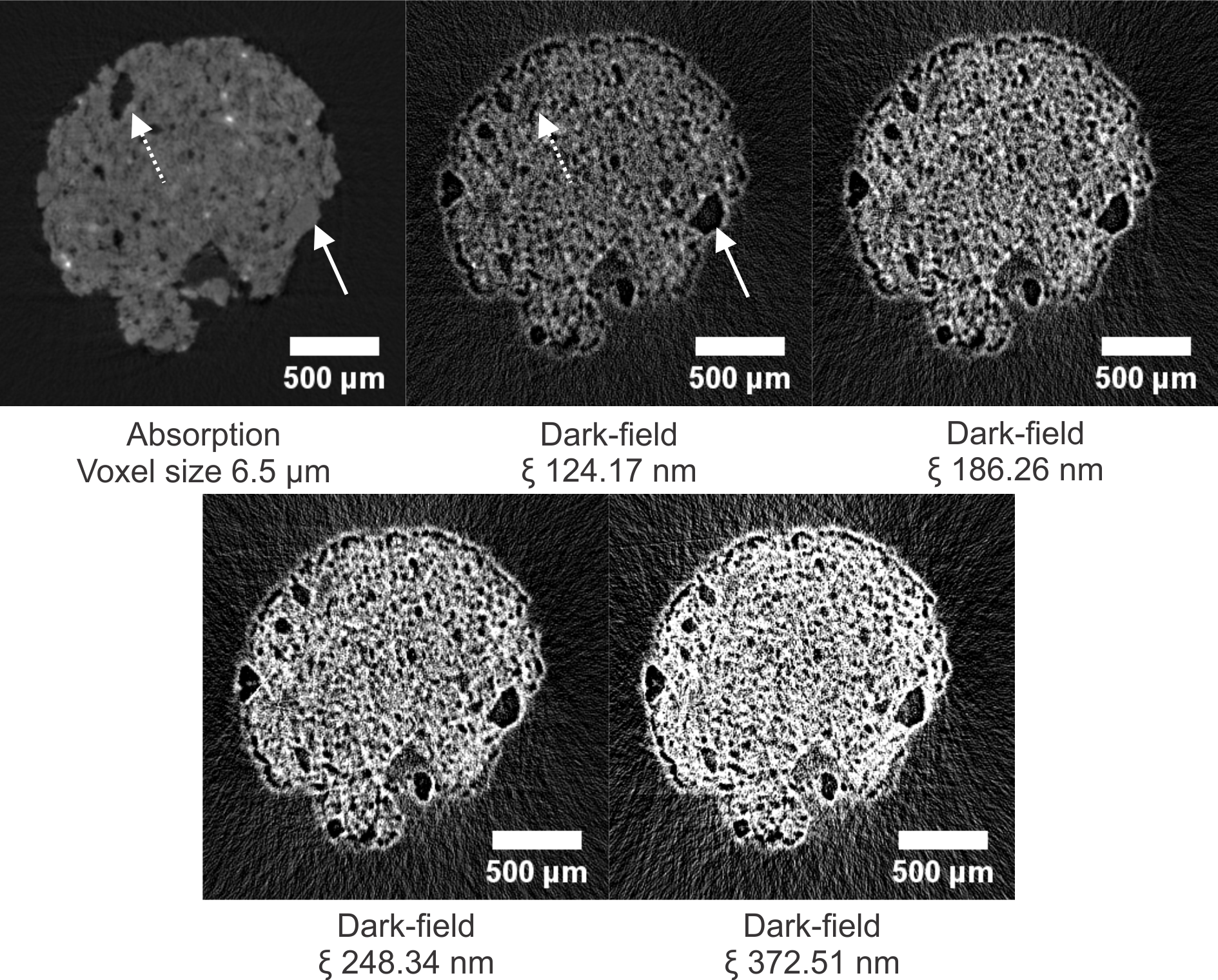

Supplement: Supplementary file 1 [file jimaging-08-00282-s001.zip › fig S1.png]

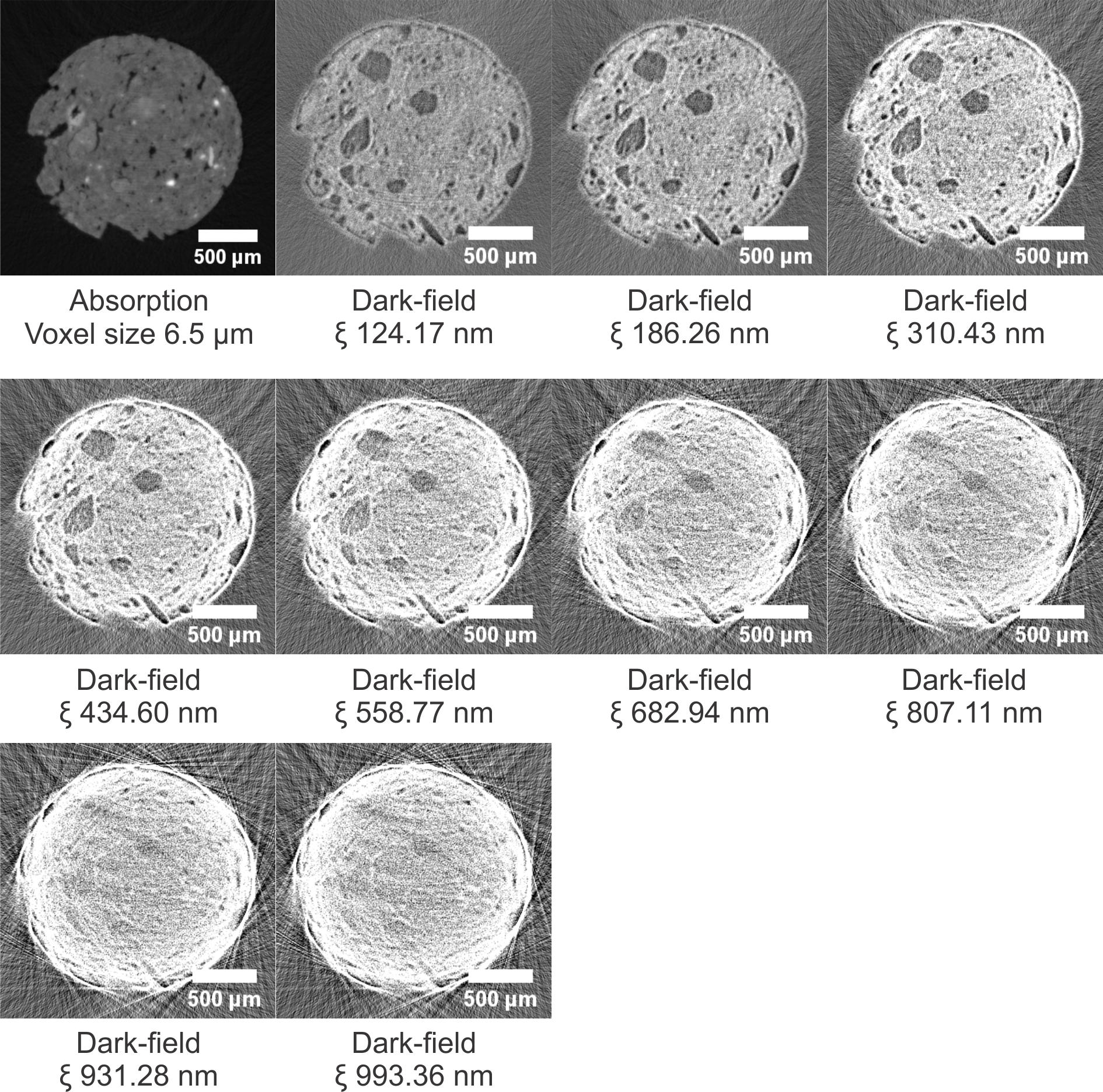

Supplement: Supplementary file 1 [file jimaging-08-00282-s001.zip › fig S2.png]

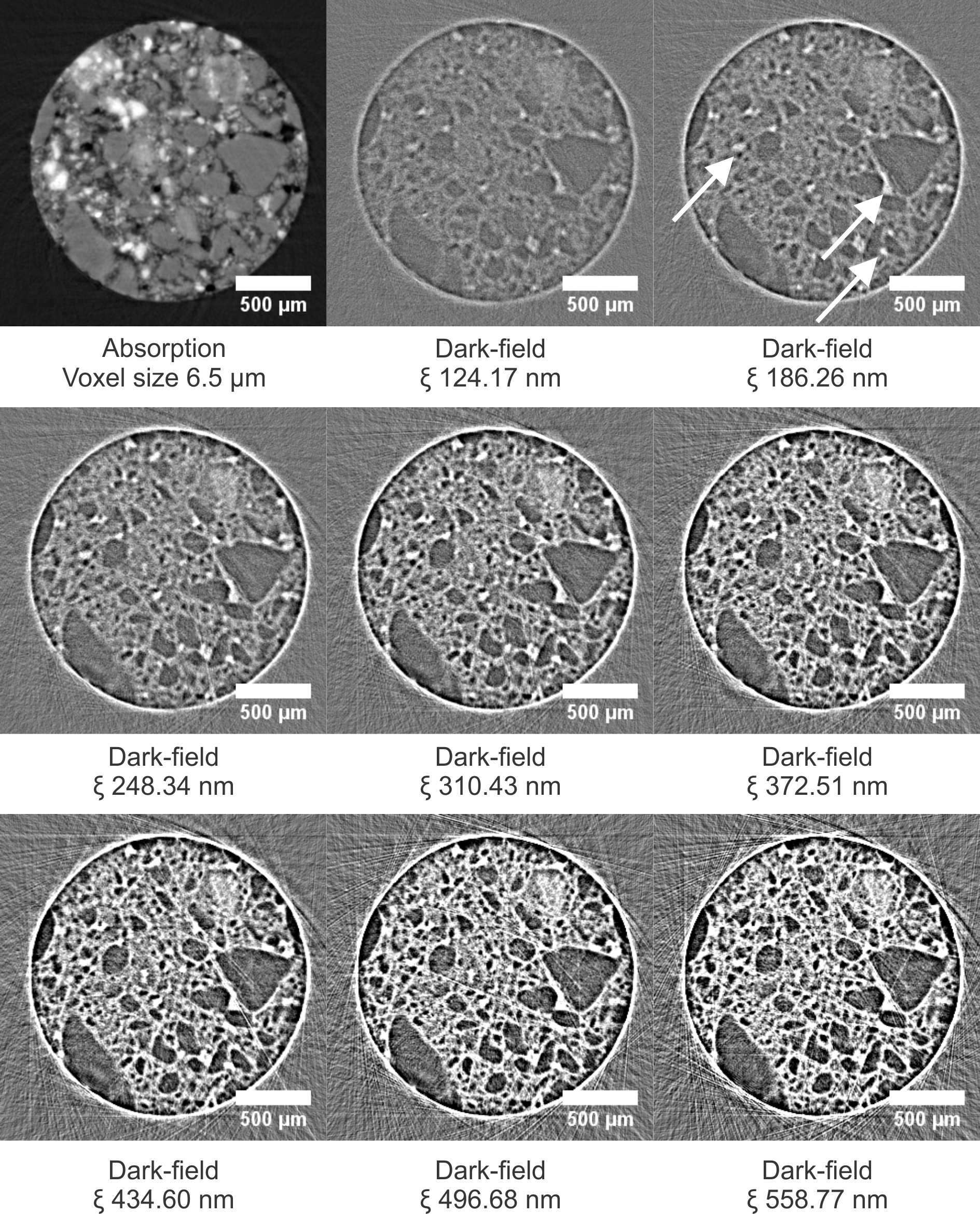

Supplement: Supplementary file 1 [file jimaging-08-00282-s001.zip › fig S3.png]
